# Structural damage in rheumatoid arthritis, psoriatic arthritis, and ankylosing spondylitis: traditional views, novel insights gained from TNF blockade, and concepts for the future

**DOI:** 10.1186/1478-6354-13-S1-S4

**Published:** 2011-05-25

**Authors:** Georg Schett, Laura C Coates, Zoe R Ash, Stefanie Finzel, Phillip G Conaghan

**Affiliations:** 1 Department of Internal Medicine 3, Institute for Clinical Immunology, University of Erlangen-Nuremberg, Krankenhausstrasse 12, 91054 Erlangen, Germany; 2 LIMM, Section of Musculoskeletal Disease, University of Leeds, Chapel Allerton Hospital, Leeds LS9 7TF, UK; 3 Section of Musculoskeletal Disease, Leeds Institute of Molecular Medicine, University of Leeds, Leeds LS9 7TF, UK; 4 Section of Musculoskeletal Disease, University of Leeds, and NIHR Leeds Musculoskeletal Biomedical Research Unit, Leeds LS9 7TF, UK

## Abstract

Structural changes of bone and cartilage are a hallmark of inflammatory joint diseases such as rheumatoid arthritis (RA), psoriatic arthritis (PsA), and ankylosing spondylitis (AS). Despite certain similarities – in particular, inflammation as the driving force for structural changes – the three major inflammatory joint diseases show considerably different pathologies. Whereas RA primarily results in bone and cartilage resorption, PsA combines destructive elements with anabolic bone responses, and AS is the prototype of a hyper-responsive joint disease associated with substantial bone and cartilage apposition. In the present review we summarize the clinical picture and pathophysiologic processes of bone and cartilage damage in RA, PsA, and AS, we describe the key insights obtained from the introduction of TNF blockade, and we discuss the future challenges and frontiers of structural damage in arthritis.

## Introduction

Structural changes of cartilage and bone resulting from arthritis were recognized in the mid-nineteenth century: witness Baker’s description of bone cysts as a protective mechanism for the joint [1]. These cysts were considered pressure-regulated escape mechanisms for the inflamed synovium into the marrow space [2]. Damage of the periarticular bone and the articular cartilage are now known to be hallmarks of arthritis, symbolizing the destructive potential of chronic inflammation. A deeper insight into the mechanism of structural changes triggered by chronic joint diseases such as rheumatoid arthritis (RA), psoriatic arthritis (PsA), and ankylosing spondylitis (AS) is essential for developing therapies that can arrest, prevent, and even reverse bone and cartilage changes. More specific interventions to treat inflammation in arthritis, for example monoclonal antibodies and soluble receptors, have added considerably to our knowledge of arthritic structural damage. In particular, the blockade of TNF has shown that effective anti-inflammatory therapy can preserve joint structure, which is critical to maintaining joint function.

RA, PsA, and AS differ substantially in their patterns of bone and cartilage damage. These differences are at least partly based on the variable capability to form new bone, which may reflect a skeletal response to inflammation. Goals and strategies to prevent and treat structural damage should therefore also differ. In the present article, we summarize the mechanistic concepts of structural damage in these three major joint diseases, we review the achievements of TNF blockers – in particular, their contribution to under standing structural damage – and we discuss unanswered questions and future frontiers in the management of bone and cartilage damage in RA, PsA, and AS.

## Rheumatoid arthritis

### Original thoughts on structural damage in RA

RA is the prototype of a destructive arthritis. The disease directly leads to joint damage, with only a few signs of repair. Tradition ally, structural damage in RA has been identified using conventional radiography to detect cortical bone erosions, joint space narrowing, and periarticular osteoporosis. Imaging has shown unequivocally that there is a net loss of cartilage and bone in patients with RA. In particular, the presence of bone erosions has emerged as an indicator of irreversible damage resulting from a continuous inflammatory attack of the synovial membrane on bone. Synovitis is of pivotal importance for bone and cartilage damage in RA. Both the severity of inflammation – whether measured by C-reactive protein, the number of swollen joints, or the duration of morning stiffness – and the duration of inflammation have therefore emerged as important predictors of structural damage in RA [3,4]. Autoantibodies such as rheumatoid factor and anti-citrullinated protein antibodies, and – in close connection to anti-citrullinated protein antibodies – the presence of the shared epitope in the HLA-DRB1 region, also predict the risk for bone erosions, which is probably related to a close association between autoantibodies and the chronicity of arthritis [5,6]. Molecularly, the tight interaction between inflammation and bone/cartilage loss in RA is explained by the production of enzymes such as aggrecanases and matrix metalloproteinases, which degrade articular cartilage and bone as well as molecules that support the differentiation of osteoclasts [7].

Bone and cartilage loss has traditionally been a main diagnostic, monitoring, and outcome parameter in patients with RA in both clinical trials and routine clinical practice. Bone and cartilage damage is rapid and dynamic after disease onset and affects the majority of RA patients within the first year [8]. The severity of bone and cartilage damage in RA is closely related to physical function in RA patients, suggesting that structural damage indeed impairs physical function [9-11]. Finally, effective control of inflammation by conventional disease-modifying anti-rheumatic drugs (DMARDs) or combination therapies of DMARDs and glucocorticoids retards structural damage in RA. Structure-sparing effects have been documented for methotrexate (MTX), sulfasalazine, and leflunomide individually and in combination [12-15]. It is not clear, however, whether MTX, sulfasalazine, leflunomide, and hydroxychloroquine directly affect bone and cartilage damage, or indirectly benefit joints by reducing inflammation.

### Novel insights gained from use of TNF blockers in RA

The introduction of TNF blockers as a therapeutic option in RA has challenged our view not only of synovitis but also of progression of structural damage. One of the most consistent effects of TNF-blocking agents in RA patients is a profound and sustained inhibition of bone erosion. In fact, all five TNF blockers approved for the therapy of RA strongly retard or even arrest structural damage [16-24]. This strong structure-preserving effect is partially due to profound and rapid control of inflammation. Also apparent, however, is that anti-resorptive effects may occur despite a lack of clinical response to a TNF blocker [24]. TNF-blocking agents thus combine a strong anti-inflammatory potential, which controls synovitis, with direct protection of bone and cartilage (Table 1).

**Table 1:** Key studies of TNF-blocker therapy in rheumatoid arthritis, 52-week follow-up

Therapy	Disease stage	Reference	Primary outcome	Radiologic outcome
Infliximab	RA	Smolen and colleagues [16]	ACR20	Modified TSS
Infliximab	Early RA	Smolen and colleagues [17]	ACR20	Modified TSS
Etanercept	Early RA	Bathon and colleagues [18]	ACR20	TSS
Etanercept	RA	Klareskog and colleagues [19]	ACR20	Modified TSS
Etanercept	Early RA	Kekow and colleagues [20]	ACR20	Modified TSS
Adalimumab	Early RA	Breedveld and colleagues [21]	ACR50	Modified TSS
Adalimumab	RA	Keystone and colleagues [22]	ACR20	Modified TSS
Golimumab	RA	Kremer and colleagues [23]	ACR50	Modified TSS
Certolizumab	RA	Keystone and colleagues [24]	ACR20	Modified TSS

ACR20/50, American College of Rheumatology 20%/50% improvement; RA, rheumatoid arthritis; TSS, total Sharp score.

In this context it is noteworthy that TNF is an important inducer of osteoclast formation and thus is a key molecular link between inflammation and bone damage [7]. Addition of TNF to monocyte cultures challenged with macrophage colony-stimulating factor and receptor activator of NF-κB ligand (molecules that activate osteoclasts, which are the cells involved in bone resorption) enhances the formation of osteoclasts, and overexpression of TNF in mice entails increased formation of osteoclasts resulting in systemic bone loss as well as local bone erosions [25-27]. With respect to cartilage damage, TNF also is an inducer of matrix enzymes such as aggrecanases and metalloproteinases, particularly MMP-1, MMP-2, and MMP-3, which are produced by synovial fibroblasts, neutrophils, and chondrocytes and degrade the cartilage matrix. A specific protective effect of TNF blockade on articular cartilage is therefore conceivable; current evidence is circumstantial, however, and is not backed by sufficient data. Direct assessment of the cartilage of small peripheral joints is still technically challenging and, to date, TNF blockers have shown little if any effect on the cartilage [28].

### Future needs and unanswered questions in RA

TNF-blocking agents have undoubtedly enriched our therapeutic options for blocking structural damage in RA. Nonetheless, several aspects remain enigmatic. The lack of adequate spontaneous joint repair and better strategies to induce joint repair will be a central field of future basic and clinical research. Indeed, any potential for erosion self-healing is still poorly characterized. Examination of sequential radiographs from clinical studies suggest that individual lesions can improve, especially when there is no or reduced swelling in the joint [29]. Other studies indicate that joint repair and erosion healing is rare despite effective therapy with TNF inhibitors [30]. More detailed imaging techniques such as ultrasound, magnetic resonance imaging (MRI), and computed tomography may provide better information in the future. Refilling bone erosions might be an important clinical goal, if the technique could restore ligament and enthesial function. If such repair proves possible, it must be followed by an assessment of joint function.

Future frontiers in RA also will include the interaction between inflammation and structural progression. With improved treatment options and tighter control of inflammation, more patients will have low disease activity or will be in remission. Even patients who are considered to be in clinical remission, however, can progress in structural damage [31-33]. How much residual synovitis is necessary to allow structural progression is not yet clear. Even subclinical synovitis may suffice to trigger a progression of cartilage and bone damage followed by a decrease in joint function. Improved detection of synovitis with ultrasound and MRI may allow a better understanding of the effect of subclinical synovitis on joint structure [34-38].

## Psoriatic arthritis

### Original thoughts on structural damage in PsA

For a long time, PsA was not recognized as a specific entity but rather was considered a subtype of RA that occurred in combination with skin psoriasis. Even after formal recognition, PsA was considered to be a mild disease with a benign course. Research in PsA has long lagged behind RA research in terms of diagnosis, prognosis, and treatment. The diagnostic criteria of Moll and Wright, although not based on patient-derived data and omitting key features of PsA such as nail disease and dactylitis, were widely used [39]. These criteria did not mitigate difficulties classifying study patients, and there fore research remained limited. The Classification Criteria for Psoriatic Arthritis now provide sensitive and specific classifications for PsA [40]. Research is still limited in early disease, however, as the Classification Criteria for Psoriatic Arthritis were built on data from patients with long-standing disease.

PsA patients suffer significant joint damage and disability over time. In accordance with RA, PsA is an erosive disease leading to the resorption of cortical bone. In addition, however, PsA shows morphological features discordant with RA; that is, the formation of bony spurs along the insertion sites of the entheses (enthesiophytes) [41]. Depending on the scoring system used, the damage and disability in PsA is less pronounced than in RA [42] or is equal to RA [43] with equivalent disease duration. Patients with RA and patients with PsA have similar functional and quality-of-life impairment [42].

Data from longitudinal cohort studies have helped identify severe disease with poor structural outcome. High inflammatory activity and joint damage at the time of presentation are considered the most important predictors of future clinical and radiologic joint damage [44,45]. For instance, a high erythrocyte sedimentation rate at baseline and the presence of joint swelling suggest a poor prognosis with respect to structural outcome [44,46]. Moreover, patients with axial disease have more severe peripheral joint disease [47].

Previously, therapies for PsA were borrowed from RA, often without any specific studies to assess their effectiveness in this different condition. There is a surprising lack of randomized controlled trials evaluating the impact of DMARD therapy on PsA. Observational studies of patients receiving traditional DMARD therapy, however, have shown little control of structural damage. Observational controlled studies with sulfasalazine and gold have shown no reduction in long-term joint damage [48,49]. An observational cohort study of 23 patients who received 2-year MTX therapy concluded that MTX treatment did not reduce radiologic progression compared with matched controls [50]. However, a more recent analysis of the same cohort – but without controls – has suggested otherwise [51]. Chandran and colleagues found that since the mid-1990s MTX had been prescribed earlier and in higher doses, resulting in a significant decline in actively inflamed joint count and psoriasis, and some decrease in progression of radiologic joint damage [51]. There is no direct evidence, however, showing that DMARD therapy affects joint damage.

### Novel insights gained from use of TNF blockers in PsA

TNF blockers have provided the first evidence-based treatment for PsA with proven effects on arthritis, skin disease, enthesitis, dactylitis, and spinal disease. These agents are highly effective in PsA, and they are the first with proven efficacy at reducing both active joint inflammation and radiographic damage in randomized con trolled trials of PsA [52-56] (Table 2). The vast majority of PsA patients treated with TNF blockers showed no worsening in radiographic damage scores [52,55-58]. Since the scoring systems used for the assessment of radiographic damage of PsA are the same as those used for RA, however, our knowledge about TNF-blocker effects on structural damage are confined to the erosive component of the disease, and it is unclear whether these agents also affect enthesiophyte formation.

**Table 2:** Key studies of TNF-blocker therapy in psoriatic arthritis

				Outcomes reported										
	
							Dactylitis		Function					
Therapy	Published study	Primary outcome	Radiologic outcomes	Joint	Skin	Nail		Enthesitis		QoL	Pain	EMS	Fatigue	CRP/ ESR
Infliximab	Antoni and colleagues [59], Kavanaugh and colleagues [52]	ACR20 at week 16	mvdH-SS at week 50	x	x		x	x	x		x			x
Infliximab	Antoni and colleagues [60], van der Heijde and colleagues [56]	ACR20 at week 14	mvdH-SS at weeks 24 and 54	x	x		x	x	x		x	x		x
Etanercept	Mease and colleagues [61]	PsARC at week 12	NA	x	x				x		x			x
Etanercept	Mease and colleagues [58]	ACR20 at week 12	mTSS at months 6 and 12	x	x				x		x			x
Adalimumab	Mease and colleagues [54]	ACR20 at week 12	mTSS at week 24	x	x		x	x	x	x			x	
Golimumab	Kavanaugh and colleagues [62]	ACR20 at week 14	NA	x	x	x	x	x	x					

ACR20, American College of Rheumatology 20% improvement; CRP/ESR, C-reactive protein/erythrocyte sedimentation rate, EMS, early morning stiffness; mvdH-SS, modified van der Heijde–Sharp score; mTSS, modified total Sharp score; NA, not available; PsARC, Psoriasis Arthritis Response Criteria; QoL, quality of life.

### Future needs and unanswered questions in PsA

The next step in investigating structural damage in PsA is to search for evidence of a direct link between inflammation and joint damage. Imaging studies have elegantly demonstrated this link in RA, using a combination of MRI, ultrasound, and conventional radiography [63,64]. Such data, however, are currently unavailable in PsA. Also of interest is the link between inflammation and new bone formation, which is typical for PsA but is not encountered in RA.

As in RA, we must evaluate the use of anti-inflammatory therapies in PsA and investigate their ability to prevent long-term damage. If there is a direct link between inflammation and damage in PsA, then tight control of inflammation may arrest damage in PsA – as has been demonstrated in RA [65,66]. Does this also apply to enthesiophyte formation? The answer is unclear and at least doubtful, as the formation of bony spurs in AS is not influenced by TNF blockade. In this context it also will be important to define treatment targets based on either clinical outcomes or imaging. For instance, a new clinical measure for minimal disease activity encompassing remission and low disease activity has been developed, but needs further testing in prospective studies [67].

## Ankylosing spondylitis

### Original thoughts on structural damage in AS

Low back pain is the earliest clinical manifestation for AS and indicates inflammation in the sacroiliac joints and the spine, which can be identified by MRI [68]. Disease progression is characterized by ongoing back pain leading to skeletal changes in the sacroiliac joints, identifiable by plain radiography. The diagnosis of AS has long hinged upon evidence of structural damage; the modified New York criteria require the presence of radiographic sacroiliitis to give a definite diagnosis [69]. Studies have shown that it can take up to 10 years for these changes to become visible on plain radiographs [70], but radiographs are still widely used in established disease. Skeletal changes in the sacroiliac joints in AS are characterized by the concomitant presence of catabolic changes such as erosions as well as new bone formation leading to progressive ankylosis.

Spinal syndesmophytes are thought to appear at a later stage [71], although this hypothesis remains unclear. This concept is supported by two facts: patients in the pre-radiographic stages of AS can suffer just as much pain and stiffness as those already diagnosed [72]; and patients treated early with TNF blockers demonstrate a better response than those treated later in their disease course [73]. Treatment should be started in the early stages of the disease process, before irreversible structural damage has occurred; that is, before the modified New York criteria are fulfilled. With the new classification criteria of spondylarthritis it will be possible to start effective medication earlier, which may yield a considerable change of the disease course in the future.

Spinal structural changes in AS are quantified radio-graphically using the modified Stoke Ankylosing Spondylitis Spine Score, which grades the cervical and lumbar spine for the presence of erosions, squaring, sclerosis, syndesmophytes, and bony bridging at each site [74]. The inability, however, of the modified Stoke Ankylosing Spondylitis Spine Score to assess the thoracic spine [75], which is the most commonly involved area as shown on MRI studies [76], limits the score’s sensitivity to assess change. Spoorenberg and colleagues have demonstrated that a minimum 2 years of follow-up is necessary to reliably detect radio graphic change, meaning that studies assessing radio graphic damage must be of significantly longer duration than similar studies in peripheral arthritis [77].

Traditionally, AS has been treated with regular physiotherapy and nonsteroidal anti-inflammatory drugs (NSAIDs). Indeed, the only evidence for a reduction in radiographic progression in patients with AS is from a trial of continuous versus on-demand treatment with NSAIDs. Patients on continuous NSAID therapy had significantly reduced radiographic progression compared with those who took the therapy only when serious symptoms were present [78]. Both groups experienced similar effects on symptoms, inflammation, and spinal mobility.

### Novel insights gained from use of TNF blockers in AS

The advent of TNF inhibitors has greatly improved the treatment options for AS. They allow treatment of patients with severe disease who do not fully respond to NSAIDs. Three TNF blockers are licensed and approved worldwide, and a fourth blocker (golimumab) was recently approved. Criteria have been set by various regulatory bodies for their use [79,80].

Similar efficacy has been found for all of the TNF blockers, although studies have consistently shown that patients relapse with cessation of therapy [81,82] (Table 3). Despite the strong anti-inflammatory effect of TNF blockers in AS, these agents do not influence new bone formation in AS [83-85]. Only one small study showed reduced radiographic progression in AS patients treated with infliximab in comparison with a historical cohort, but these results have to be considered with caution as the baseline Bath Ankylosing Spondylitis Disease Activity Index score was different between the groups [86,87].

**Table 3:** Key studies of TNF-blocker therapy in ankylosing spondylitis

Outcomes reported											
**Therapy**	**Published study**	**Primary outcome**	**Radiologic outcome**	**Follow-up**	**BASDAI**	**BASFI**	**BASMI**	**QoL**	**EMS**	**Fatigue**	**CRP/ ESR**

Infliximab	Braun and colleagues [96-100]	50% improvement in BASDAI at week 12	mSASSS	5 years	x	x	x	x	x		x
Infliximab	Marzo-Ortega and colleagues [101]	Change in BASDAI at weeks 4, 10, 30	MRI inflammatory lesions	30 weeks	x	x		x	x		x
Infliximab	van der Heijde and colleagues [83,102]	ASAS 20 at week 24	mSASSS	8 years	x	x	x	x	x	x	x
Etanercept	Davis and colleagues [103-105], van der Heijde and colleagues [84]	ASAS 20 at week 12	mSASSS	16 years	x	x	x		x		x
Etanercept	Calin and colleagues [106], Dijkmans and colleagues [107]	ASAS 20 at week 12	mSASSS	8 years	x	x	x		x		x
Adalimumab	van der Heijde and colleagues [108]	ASAS 20 at week 12	NA	24 weeks	x	x	x	x	x		x
Golimumab	Inman and colleagues [109]	ASAS 20 at week 14	NA	24 weeks	x	x	x	x	x		x

ASAS, Assessment of Spondyloarthritis International Society; BASDAI, Bath Ankylosing Spondylitis Disease Activity Index; BASFI, Bath Ankylosing Spondylitis Functional Index; BASMI, Bath Ankylosing Spondylitis Metrology Index; CRP/ESR, C-reactive protein/erythrocyte sedimentation rate; EMS, early morning stiffness; MRI, magnetic resonance imaging; mSASSS, modified Stoke Ankylosing Spondylitis Spine Score; NA, not applicable; QoL, quality of life.

This lack of a structure-sparing effect of TNF blockers in AS unravels the different pathophysiologic mechanisms underlying RA, PsA, and AS. RA is typically characterized by bone erosion, whereas the main structural out come in AS is bony spur formation based on bone formation.

Radiographic damage at baseline is a major predictor of future radiographic progression; in particular, the presence of syndesmophytes or ankylosis [86,88]. MRI-evident sacroiliitis and positivity for HLA-B27 have been shown to predict the development of radiographic sacroiliitis in patients with early inflammatory back pain at 8-year follow-up, with a sensitivity and specificity of 77% each [89]. MRI has now been incorporated into the new Assessment of Spondyloarthritis International Society classification criteria for axial spondyloarthritis [90].

The relationship between inflammation and new bone formation in AS remains unclear. Recent MRI studies have shown that active corner inflammatory lesions (also known as Romanus lesions or bone edema) predict the development of syndesmophytes [91]. These studies also demonstrated formation of syndesmophytes at the exact location of resolved inflammatory lesions. One explanation is that there may be persistent mild inflammation not detected by MRI. The discovery that syndesmophytes were more likely to develop at the sites of resolved corner inflammatory lesions rather than those of persistent lesions, however, led to the TNF brake hypothesis. This hypothesis suggests that TNF triggers pathways leading to new bone formation, but that while there is active inflammation TNF suppresses new bone formation via dickkopf-1 (a regulator of joint remodeling) [92]. When patients are treated with TNF inhibitors, therefore, inflammation resolves, the brake is released, and tissue repair and new bone formation occur [91]. This process may account for radio graphic progression in patients who appear to otherwise respond well to anti-TNF therapy. Evidence for uncoupling between inflammation and new bone formation is supported by a mouse model of spondyloarthritis, which showed no effect of etanercept on the severity and incidence of joint ankylosis [93].

An independent study of patients receiving anti-TNF agents found that an inflamed vertebral edge at baseline had a threefold increased risk to develop a syndesmophyte than a non-inflamed vertebral edge [94]. These results contrast with those of Maksymowych and colleagues [91]. MRI scans were performed only at baseline and 2 years, however, so it is possible that inflammation had occurred and resolved between scans. Bennett and colleagues described fatty Romanus lesions in the spine, which they suggest may be the post-inflammatory phase between osteitis on MRI and sclerotic bone formation on radiographs [95].

## Future needs and unanswered questions in AS

Despite the efficacy of TNF blockers for symptomatic control and improved quality of life in patients with AS, the lack of efficacy for radiographic progression is noteworthy. Longer studies may be needed, because the process linking inflammation and new bone formation is slow. Effective suppression of inflammation may thus still reduce radio graphic progression.

Additional research is needed to analyze whether progression is due to persistent, low-grade inflammation or to the release of the TNF brake once inflammation is effectively treated. The answer to this question will guide future therapies. Perhaps a dual approach will be necessary: one therapy to treat inflammation and another to prevent new bone formation.

MRI has facilitated the study of early disease, and the treatment response of patients in the pre-radiographic stage may help determine whether suppression of inflammation can prevent early onset of structural damage. Since not all patients with MRI-evident sacroiliitis develop AS, however, treatment must be carefully targeted. Lastly, there is the issue of late presentation of AS patients to rheumatologists, which can be improved by the education of both doctors and patients.

## Abbreviations

AS: ankylosing spondylitis; DMARD: disease-modifying antirheumatic drug; MMP: matrix metalloproteinase; MTX: methotrexate; MRI: magnetic resonance imaging; NF: nuclear factor; NSAID: nonsteroidal anti-inflammatory drug; PsA: psoriatic arthritis; RA: rheumatoid arthritis; TNF: tumor necrosis factor.

## Competing interests

ZRA has received research grants from Schering-Plough Corporation, now Merck Sharp & Dohme. PGC has received research grants from Pfizer, and has been a speaker for Merck & Company, Inc. and AstraZeneca. The remaining authors declare that they have no competing interests.
